# Prediction of the Glass Transition Temperature in Polyethylene Terephthalate/Polyethylene Vanillate (PET/PEV) Blends: A Molecular Dynamics Study

**DOI:** 10.3390/polym14142858

**Published:** 2022-07-13

**Authors:** Mattanun Sangkhawasi, Tawun Remsungnen, Alisa S. Vangnai, Phornphimon Maitarad, Thanyada Rungrotmongkol

**Affiliations:** 1 Program in Biotechnology, Faculty of Science, Chulalongkorn University, Bangkok 10330, Thailand; mattajung@gmail.com; 2 Faculty of Interdisciplinary Studies, Khon Kaen University, Nong Khai Campus, Nong Khai 43000, Thailand; 3 Center of Excellence in Biocatalyst and Sustainable Biotechnology, Department of Biochemistry, Faculty of Science, Chulalongkorn University, Bangkok 10330, Thailand; alisa.v@chula.ac.th; 4 Research Center of Nano Science and Technology, Shanghai University, NO 99, Shangda Road, P.O. Box 111, Baoshan district, Shanghai 200444, China; pmaitarad@shu.edu.cn; 5 Program in Bioinformatics and Computational Biology, Graduate School, Chulalongkorn University, Bangkok 10330, Thailand

**Keywords:** polyethylene terephthalate, polyethylene vanillate, blend polymer, glass transition temperature, molecular dynamics simulation

## Abstract

Polyethylene terephthalate (PET) is one of the most common polymers used in industries. However, its accumulation in the environment is a health risk to humans and animals. Polyethylene vanillate (PEV) is a bio-based material with topological, mechanical, and thermal properties similar to PET, allowing it to be used as a PET replacement or blending material. This study aimed to investigate some structural and dynamical properties as well as the estimated glass transition temperature (*T_g_*) of PET/PEV blended polymers by molecular dynamics (MD) simulations with an all-atom force field model. Four blended systems of PET/PEV with different composition ratios (4/1, 3/2, 2/3, and 1/4) were investigated and compared to the parent polymers, PET and PEV. The results show that the polymers with all blended ratios have *T_g_* values around 344–347 K, which are not significantly different from each other and are close to the *T_g_* of PET at 345 K. Among all the ratios, the 3/2 blended polymer showed the highest number of contacting atoms and possible hydrogen bonds between the two chain types. Moreover, the radial distribution results suggested the proper interactions in this system, which indicates that this is the most suitable ratio model for further experimental studies of the PET/PEV polymer blend.

## 1. Introduction

The study of polymer blends by mixing two or more polymers as a strategy for developing the properties of a polymer has been continuously reported for a decade. It is simple to create materials with enhanced qualities by combining their excellent properties. Polyethylene terephthalate (PET) is an essential engineering thermoplastic polyester used in a broad range of applications, especially bottles for beverages and food packaging [1]. However, PET is a non-biodegradable product that requires a long time to decompose, and its accumulation in the environment is both a direct and indirect health risk to humans and animals [2]. The blending of PET with nanocomposite, composite, thermoplastic, and biodegradable polymers [3] is of interest to reduce its use and decomposition time.

**Figure 1:**
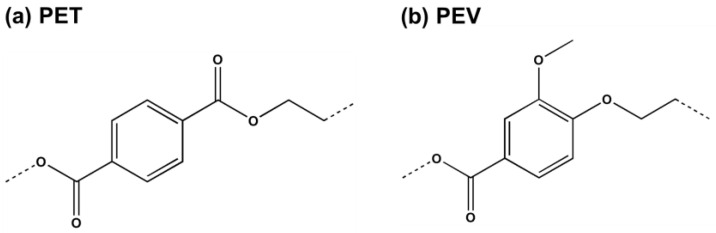
Two-dimensional structures of the two single-chain polymers: (**a**) polyethylene terephthalate (PET or T) and (**b**) polyethylene vanillate (PEV or V) for PET/PEV blends.

Bio-based and biodegradable polymer blends with PET have been reported, such as poly (butylene succinate) (PBS) [4], poly(lactic acid) (PLA) [5,6,7,8], polyamide56 [9], and poly(ethylene 2,5-furanoate) (PEF) [10], which presented an improvement in the thermal stability and/or mechanical properties. Interestingly, a bio-based polymer called polyethylene vanillate (PEV) was found to have thermomechanical and structural properties similar to PET [11,12]. Their chemical structures are shown in Figure 1. PEV is produced by vanillic acid (VA) as a precursor and has good potential as a bio-based alternative to PET [11]. Remarkably, PEV could be an excellent choice to blend with PET to improve the properties of the polymer and make this new material more eco-friendly. The PET/PEV blends have not been experimentally tested yet. Therefore, this study is the first report to use a computational method to investigate the structure, dynamics, and thermal properties of PET/PEV blends in different ratios. The obtained information could help to guide the synthetic chemists who are interested in the development and use of PET/PEV blends.

One important key to defining the use of polymer is its glass transition temperature (*T_g_*) [13,14], which can be estimated and predicted by computational methods such as molecular dynamics (MD) simulation [15,16,17,18,19] via some thermodynamic properties such as the specific volume [13,19,20]. This study aimed to estimate the *T_g_* and evaluate some structural and dynamical properties of PET/PEV blends at different ratios using all-atom MD simulations with the force field parameters obtained from the previous study [21].

## 2. Materials and Methods

### 2.1. Model and Parameter Preparation

The PEV (0/5) and PET (5/0) polymers and their four blended PET/PEV ratios, i.e., 1/4, 2/3, 3/2, and 4/1, were prepared and named as TV05, TV50, TV14, TV23, TV32, and TV41, respectively. The six systems are summarized in Table 1. Every single chain of PET (T) and PEV (V) consisted of 100 optimized repeating units. Their atomic partial charges were taken from our previous study [21], while the parameters were based on the general force field OPLS-AA [22,23]. All five single chains of PET and/or PEV were first placed in parallel in a cubic box length of ~1500 Å. This length was then reduced along with the simulation driven by a force field at high temperature and pressure of 600 K and 250 atm, respectively, to an approximated simulation equilibrium box length.

### 2.2. Molecular Dynamics Simulations

All six systems were built by all-atom MD simulations with the time step of 1.0 fs using DLPOLY version 4.0 [24]. The Berendsen thermostat and barostat with relaxation times of 1.0 ps were applied to control system temperature and pressure under the NPT ensemble, respectively. The cut-off for long-range and short-range interactions is 12.0 Å with the shifted-force potential method. Each simulation is separated into three steps. Firstly, the system was run through MD simulation under an NPT ensemble with high temperature at 600 K and high pressure at 250 atm for 1 ns. The high pressure was applied to accelerate polymer folding, resulting in the amorphous polymer. The PET/PEV chains were compressed dramatically in the simulation box. The final configuration was then used as the initial structure for each system. Secondly, the systems were then equilibrated step by step from 600 K to 100 K, with an interval of 100 K at 1 atm for 10 ns. Each subsequent simulation was started from the 2 ns point of the adjacent equilibration run at the higher temperature. Lastly, further production runs of 10 ns were carried out, and the MD trajectories were saved every 1.0 ps (1000 steps) for evaluating structural and dynamical properties.

### 2.3. Structural Characterization

The conformations of the PET/PEV blend polymer in the production phase of 10 ns at 300 K were extensively analyzed. The aromatic carbon, ethylene carbon, carbonyl carbon, oxygen, ester oxygen, and ether oxygen are labeled as CA, CT, CO, O, OES, and OS, respectively, for both PET and PEV. For discussion of the compatibility between PET and PEV polymer chains, the site–site RDFs, describing the chain folding of blend polymer from the CA to the CT, CO, OES, and O atoms, and the CA–CA atom pair were calculated by the Visual Molecular Dynamics (VMD) program version 1.9.3 [25]. The number of atoms in contact, the number of hydrogen bonds between polymer chains, and the radius of gyration of each polymer were plotted and compared. Lastly, the orientation correlation function of benzene rings and the dihedral angle distribution of PET/PEV blends relative to PET and PEV polymers were also discussed.

## 3. Results and Discussion

### 3.1. Folding of PET/PEV Blends

**Figure 2:**
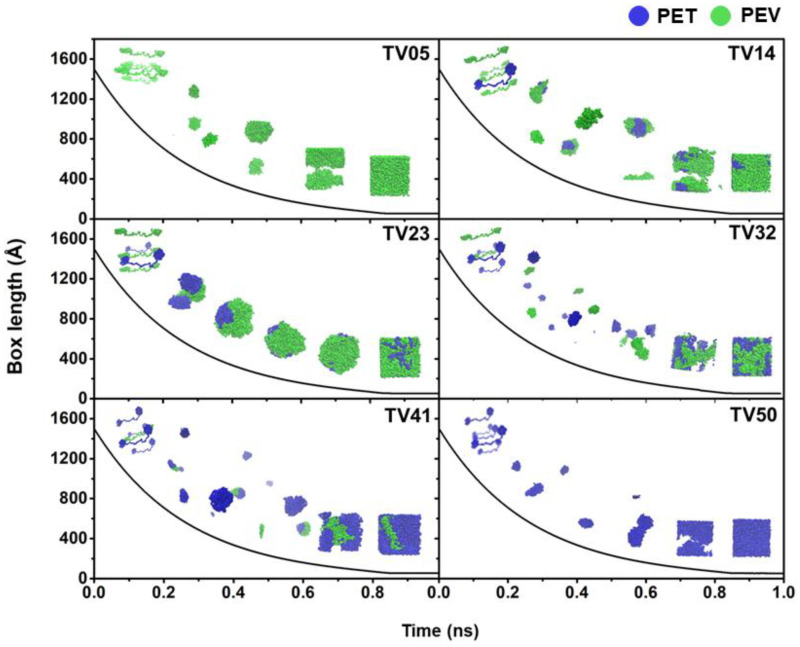
The folding conformations of PET, PEV, and their blend polymers along simulation time of 1.0 ns at 600 K and 250 atm.

**Figure 3:**
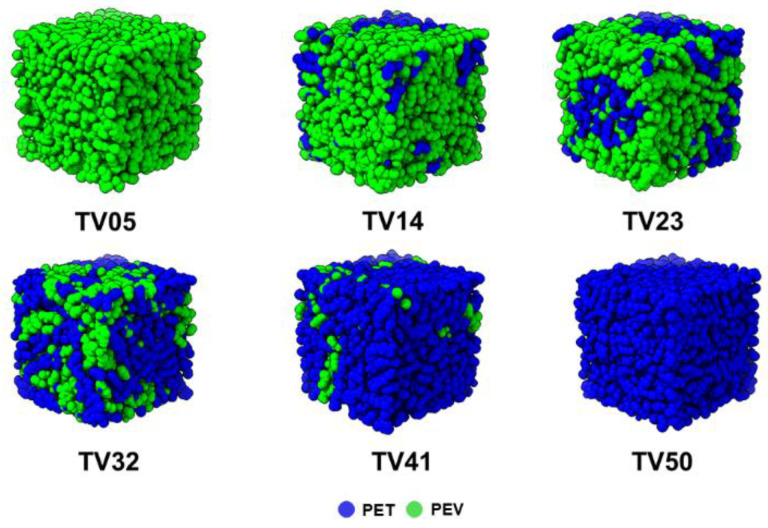
Simulated morphologies of PET, PEV, and PET/PEV blends at different composition ratios. The blue and green colors represent PET and PEV, respectively.

At the beginning of a simulation at 600 K and 250 atm, all five long linear chains of PET and/or PEV were placed parallel in a large enough cubic box of ~1500 Å in length. Figure 2 shows that the box lengths of all simulated systems dramatically decreased within the first 0.85 ns, while the morphology of the PET, PEV, and blended systems at 1.0 ns are depicted in Figure 3. At the end of the 1.0-ns MD simulations, the obtained box lengths were approximately 52.8, 52.6, 52.4, 52.2, 52.0, and 51.7 Å for TV05, TV14, TV23, TV32, TV41, and TV50, respectively. The most extended box length was found for TV05 in accordance with the higher total atoms in the pure PEV system, and the box length decreased as the proportion of PET increased. The last configuration was used as the starting structure of the simulation at 600 K and 1.0 atm for 10-ns equilibration and 10-ns production runs. The simulations at lower temperatures were conducted step by step with 100 K intervals to 100 K. Each following simulation was started from the 2 ns point of the adjacent equilibration run.

In the 10-ns production phase at 100–600 K, the total energy, temperature, coulombic energy, van der Waal energy, and end-to-end distance of each polymer chain were plotted versus simulation time and are presented in Supplementary Figure S1. All results with low fluctuation across the time suggest that the MD trajectories from the production phase can be used for further analysis and discussion in the following sections.

### 3.2. Glass Transition Temperatures

**Figure 4:**
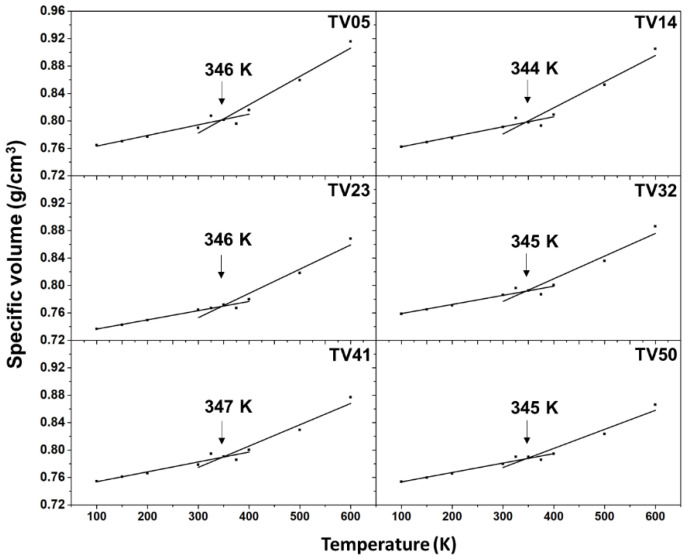
Temperature dependence of the specific volume for PET, PEV, and PET/PEV blends. The intersection of the two trended lines from low to high temperatures defines the *T_g_* of the polymer.

Some previous works have reported the prediction of the *T_g_* of pure and blended polymers by MD simulations [16,19,20]. The specific volumes of each PET/PEV blended system from the final simulation box length were plotted versus the temperatures from 100 K to 600 K relative to the PET and PEV (Figure 4). The predicted *T_g_* of each system was the temperature at the intersection between the two trending lines that fitted from low temperatures of 100–300 K to high temperatures of 400–600 K. It should be noted that the *T_g_* prediction by monitoring the specific volume or density of a system per temperature has been used successfully in several polymers [13,14,20,21,26,27].

The results in Figure 4 show *T_g_* values of 344–347 K for PET/PEV blends, in the same range for PEV (346 K) and PET (345 K), and which are close to the experimental [11,12,28] and computational [21,29] studies (Table 2). The *T_g_* values for all PET/PEV ratios were not significantly different, unlike the PMMA/PS blends, which showed an increase in *T_g_* as the percentage of PS was enhanced [19]. Increased PEV chains in the simulation model in the present study slightly decreased the predicted *T_g_* value compared to the single-chain polymer [21,29]. This was not found in the case of PET. Based on the predicted *T_g_*, the blended PET/PEV polymers can be used for various applications, similar to PET. Nonetheless, the synthesis of PET/PEV blending is required to validate our *T_g_*-predicted model.

### 3.3. Mean Square Displacements

**Figure 5:**
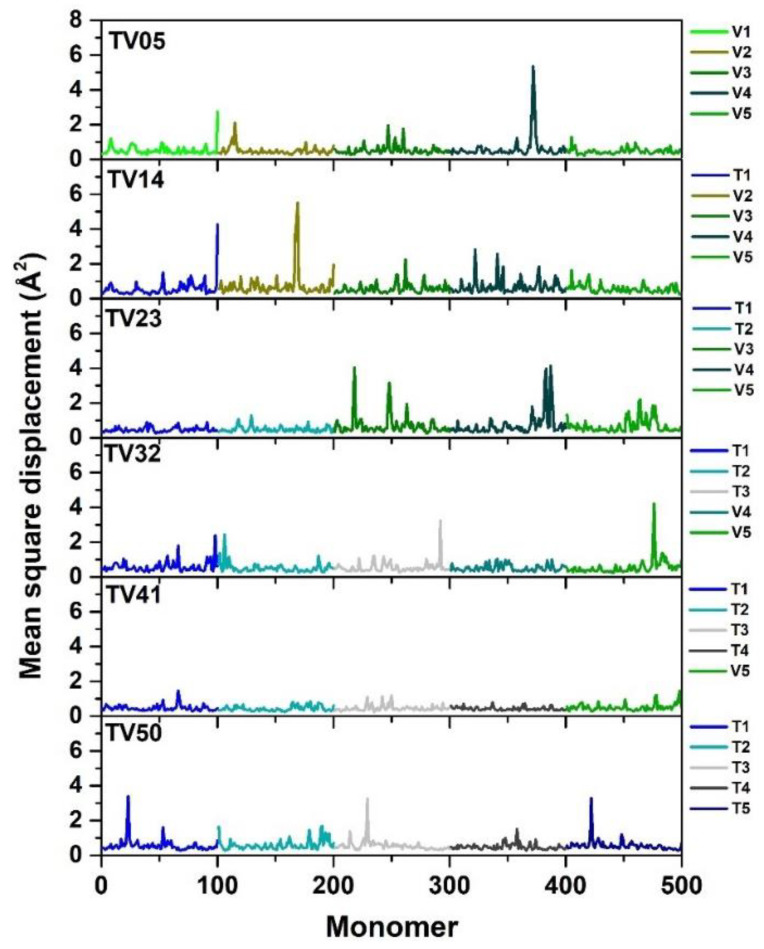
The mean square displacement of the PET/PEV blend system for the 5 ns. Each system consisted of 5 single chains of PET and/or PEV represented by T and V, respectively.

To evaluate the system’s flexibility, the mean square displacement (MSD) was used to explain the motion of monomers along a chain [18,20]. The center-of-mass MSDs of every monomer in all chains were obtained, including 500 monomers, and are shown in Figure 5. The PET (TV50) chains are a bit more rigid than the PEV (TV05) chains. A more rigid structure of PET was also mentioned in previous work [21]. The MSD results show a high peak at chains T1, V2, and V3 in TV14 and V3 and V4 in TV23, indicating that some of the chains in this ratio may move flexibly. This means they may not interact with other chains in this section. In agreement with TV32 and TV41, which gave MSD results less than PEV but close to PET, this implies that both of these ratios may fold similarly to PET. Moreover, when focusing on the blended system at 5 ns, the MSD results of TV41 show the most rigid system, followed by TV32, TV23, and TV41. It may be concluded that the blending proportionality between PET and PEV affects the stiffness and elasticity of the blended polymers. As the ratio of the PET chains increases, so does the system’s rigidity. The MSD results at 2.0, 5.0, and 10 ns are plotted in Figure S2, which also shows the same trend.

### 3.4. Structural Properties of Folded Polymer

#### 3.4.1. The Site–Site Radial Distribution Functions

**Figure 6:**
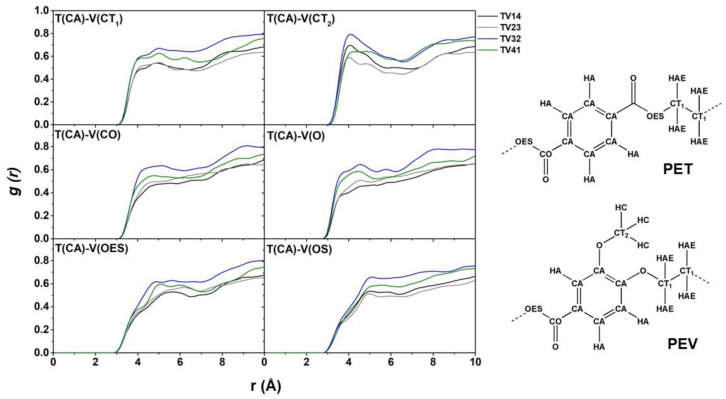
The site–site RDFs, *g(r)*, of PET chains are centered on the CA atom of the polymer benzene ring and extend to the CT ethylene carbons and the ester components CO, O, and OES of PEV chains.

**Figure 7:**
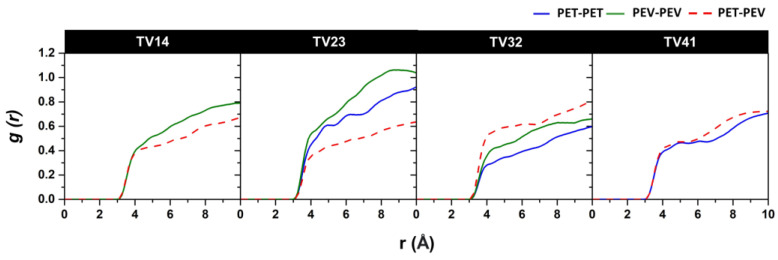
The site–site RDFs of CA–CA atom pairs between the polymer benzene rings of four studied PET/PET blends.

The radial distribution function (RDF), *g(r)*, is a probability function that finds particles at different distances from a given particle and is helpful in the structural analysis [18,30,31]. The *g(r)* of the atom pair between the PET and PEV chains was calculated for the blended systems. Figure 6 demonstrates the *g(r)* between T(CA) of the PET chains and V(CT), V(CO), V(O), V(OES), and V(OS) of the PEV chains. Among the four blended polymers, the TV32 system has significantly higher RDF peaks in all pairs, indicating strong interactions between PET and PEV polymers. The peaks with oxygen sites of PEV, i.e., OS, OES, and OS, were detected before the carbon sites. In addition, the site–site RDFs of the CA–CA atom pair of PET–PEV chains were calculated and are shown in Figure 7. The *g(r)* peak of PET–PEV (dashed line) is higher than the *g(r)* peaks of all PET–PET and PEV–PEV (solid line) systems at approximately 3–4 Å in TV32 and TV41. The finding indicates the strong interaction between the different types of PET–PEV polymers, these being greater than those of the PET–PET and PEV–PEV polymers themselves, in particular TV32. This implies that PET is likely to be miscible with PEV throughout the composition in a blended PET–PEV ratio of 60:40. In contrast, for TV14 and TV23, the dashed line is lower than the solid line, referring to polymer immiscibility or the composition located in the miscibility gap. The predicted miscibility of all blended systems is ranked as follows: TV32 > TV41 > TV14 > TV23. This phenomenon was found in blended PET/PLA systems [5], in which PET–PLA’s *g(r)* is higher than those of PET–PET and PLA–PLA in all blended ratios indicating miscible properties. Furthermore, *g(r)* was used to determine the degree of miscibility of the polymer blend in the PLA/PEG study [32].

#### 3.4.2. Interactions between Polymer Chains

**Figure 8:**
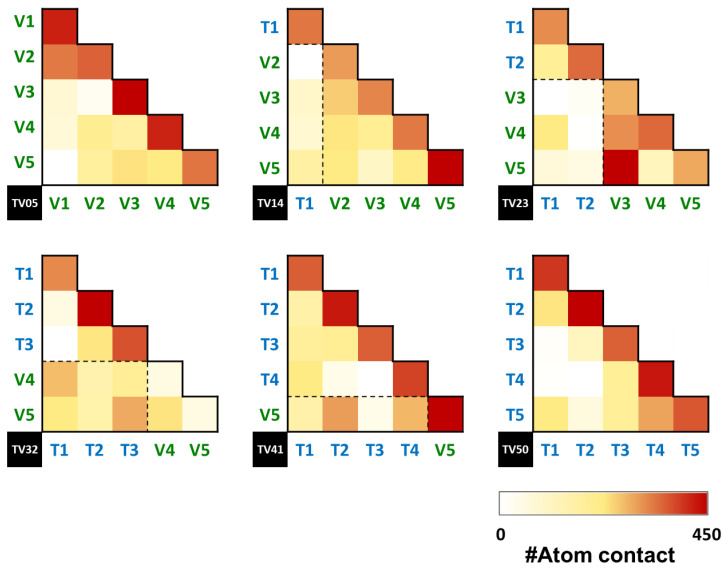
The heatmap of the number of contact atoms between each chain of the PET, PEV, and PET/PEV blends.

The number of contact atoms between PET and PEV chains within 6 Å is plotted along with the simulation time in Figure S3. The TV32 system showed likely higher contacting atoms than others as in the following order: TV32 (1197.52 ± 31.39) > TV23 (941.45 ± 27.87) > TV41 (730.83 ± 25.13) > TV14 (610.04 ± 22.18). In detail, the number of contact atoms between the same type of polymer (PET–PET and PEV–PEV) and a different type of polymer (PET–PEV in dashed box) is shown as the heatmap in Figure 8. The TV32 and TV41 systems had more favorable contacts between PET and PEV chains for T1–V4 (237.2) and T3–V5 (251.7) in the TV32 system and T2–V5 (259.6) in the TV41 system.

**Figure 9:**
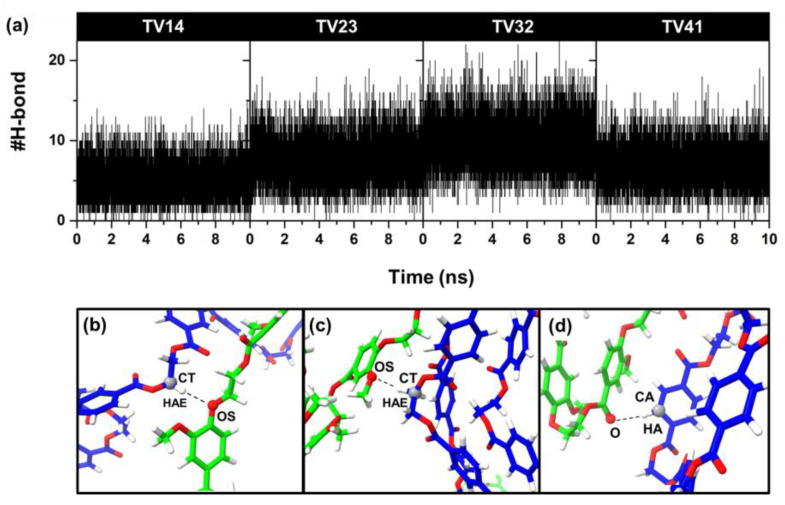
(**a**) The number of hydrogen bonds formed between PET and PEV chains per time. (**b**–**d**) Examples of hydrogen bonds between PET (blue) and PEV (green).

The hydrogen bond is an essential interaction in macromolecular systems, including polymers. Herein, the number of possible hydrogen bonds formed between the PET and PEV chains was measured with a cut-off of 3.5 Å for CH…O and an angle of 180 ± 20 degrees for CH-H…O. The plot of hydrogen bonding per time and some examples of hydrogen interaction between PET and PEV chains are illustrated in Figure 9. The CH group can act as a proton donor [33,34], similar to conventional hydrogen bonds in most ways. It was found that oxygen atoms such as OS and O in the PEV chains prefer to form hydrogen bonds with the hydrogen of carbon atoms such as CT and CA in the PET chain. As expected, the TV32 system revealed the highest number of hydrogen bond interactions between two types of polymers (9.8 ± 3.0), followed by TV23 (7.4 ± 2.7), TV41 (6.8 ± 2.5), and TV14 (5.1 ± 2.2). However, these hydrogen bonds between the two different polymers are rather weak, as seen in a significantly reduced number of hydrogen bond interactions in Figure S4 using a cut-off of 3.0 Å for CH…O.

**Figure 10:**
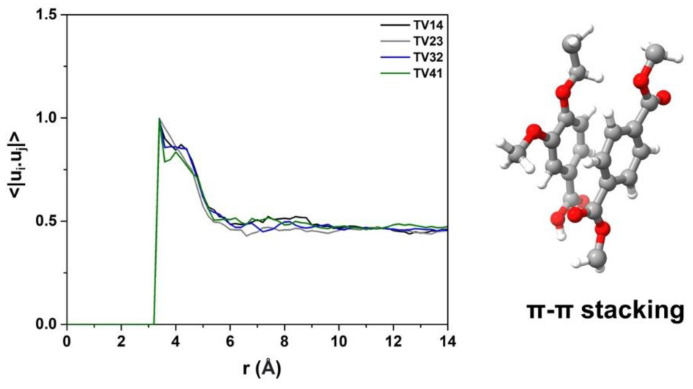
The orientation correlation function of the benzene ring in different blend ratios of PET/PEV.

Because the RDF results in Figure 7 cannot fully designate the packing structure of the benzene ring between different polymer chains, the orientation correlation function (OCF) of the benzene ring was calculated using <|u_i_.u_j_|>. u_i_ and u_j_ represent the unit vectors perpendicular to the plane of the benzene ring in PET chains and PEV chains, and r is the distance between the centers of mass of two considerable rings. When the value of <|u_i_.u_j_|> is close to 1.0, the two benzene rings are almost parallel [18]. As shown in Figure 10, the OCFs of different PET/PEV blended systems are similar, with a high peak at ~ 3.4 Å, indicating the parallel displacement of two rings.

#### 3.4.3. Radius of Gyration of Two Blends

**Figure 11:**
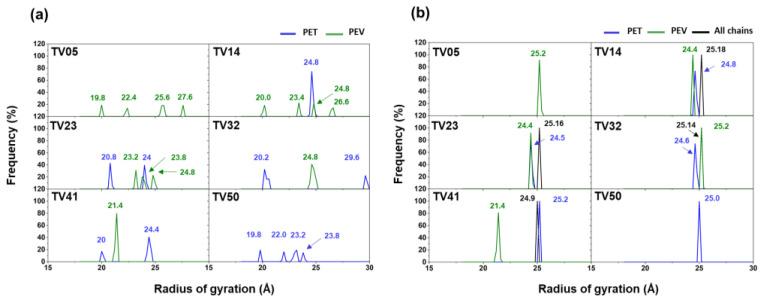
The radius of gyration of PET and PEV, and PET/PEV blends; (**a**) shows the distribution of *R_g_* of each five chains in the systems, while (**b**) shows the distribution of *R_g_* of each polymer type PET (blue), PEV (green), and all chains (black).

The radius of gyration (*R_g_*) of blended polymers was previously studied to compare the miscibility of PPE, PS, and PMMA polymers [18]. The distribution of *R_g_* for PPE–PMMA is significantly broader with increasing chain lengths of PPE to 30 monomers, i.e., the wider distribution of the PPE’s *Rg* could present the immiscible system. Thus, the *R_g_* of PET and PEV in the present study of PET/PEV blended systems with different ratios are considered and compared in Figure 11. The Rg of the five chains in PET, PEV, and PET/PEV blend systems is shown in Figure 11a, which presents the range of *R_g_* values at 19 Å and 28 Å for TV05 and 19 Å to 24 Å for TV50. The distribution of *R_g_* in TV14, TV23, and TV41 is similar to that of TV05 and TV50, with the exception of TV32, which has the *R_g_* of the PET chain at 29.6 Å due to free polymer folding that can occur in some polymer chains. Figure 11b depicts the Rg of each polymer type PET (blue), PEV (green), and all chains (black). The *R_g_* of all chains in TV14 (25.18 Å), TV23 (25.16 Å), TV32 (25.14 Å), and TV41 (24.9 Å) are not significantly different from TV05 (25.2 Å) and TV50 (25.0 Å), indicating that PET/PEV blend systems are miscible. Furthermore, the *R_g_* of all chains in TV32 and TV41 is slightly lower than the *R_g_* of all chains in TV05, the traditional PET polymer. The discovery of a separated *R_g_* of PEV value (21.4 Å) in TV41 was caused by the presence of a single PEV chain that comprised the TV41 system. However, when the *R_g_* of each chain is focused on in Figure 11a, the isolated peak of PEV remains in the same range as the *R_g_* of five PEV chains in the TV05 system, which has no effect on TV41 miscibility.

## 4. Conclusions

PEV is being considered as a PET replacement or blend to reduce PET accumulation in the environment. The aim when blending PET/PEV is to find the appropriate blend ratio that is best suited for experimental testing. Even though PEV is not yet a commercial product, blending PET/PEV can produce a more easily degradable material in the environment since PEV is a bio-based polymer from lignocellulosic biomass. We also intend to eventually replace PET with PEV. Blending is a step toward this objective. Computational models and simulations could predict some properties of blended polymer systems, reducing the investigation cost and avoiding much experimental work. In this study, MD simulations using the all-atom OPLS-AA force field were used to determine the structural and dynamical features of PET/PEV blends, including molecular folding, configurations, and *T_g_*. High temperature and pressure can fold and orient long linear polymer chains into a simulation box, which could be used as a starting configuration for additional simulations. The equilibrium box lengths or volumes for each temperature in the NPT ensemble are used to predict *T_g_*. Since the obtained *T_g_* for all blended systems is not significantly different, ranging from 344 to 347 K, these are not far from previous computational and experimental values of the parent polymers, PET and PEV. The blended PET/PEV polymers may have a wide range of applications, similar to PET. The preservation of the molecular structures of monomers in both the PET and PEV polymers resulted in a similar pattern for structural characterization with site–site RDFs. Furthermore, TV32 had the highest contact atom number, *g(r)*, and number of hydrogen bonds between PET and PEV. Overall, the obtained structural and dynamical properties are not significantly different. Finally, all the results suggest that the TV32 system, which responds to a PET–PEV ratio of 60:40 is attractive for uses similar to PET.

## Figures and Tables

**Table 1:** Simulation details of six systems: PEV, PET, and their blends with different ratios.

System	PET/PEV Compositions	Number of PET Chains	Number of PEV Chains	Number of Atoms
TV05	0/100	0	5	12,015
TV14	20/80	1	4	11,815
TV23	40/60	2	3	11,615
TV32	60/40	3	2	11,415
TV41	80/20	4	1	11,215
TV50	100/0	5	0	11,015

**Table 2:** Glass transition temperature (*T_g_*) of PET, PEV, and PET/PEV blends predicted from our MD simulations compared to the previously reported theoretical and experimental data.

	Glass Transition Temperature (K)					
	PET (TV50)	PEV (TV05)	TV14	TV23	TV32	TV41
MD simulation	345, 345 [21], 342 [12]	346, 353 [21]	344	346	345	347
Experiment	353 [12], 350 [29]	347 [11], 348 [28], 356 [12]	NA			

## Data Availability

All the data generated for this publication have been included in the current manuscript.

## Supplementary Materials

The following supporting information can be downloaded at: https://www.mdpi.com/article/10.3390/polym14142858/s1, Figure S1: The investigation of system equilibrium (a) box length (Å) of the production run from 1 to 10 ns, (b) temperature, (c) total energy profile (kcal/mol), (d) coulombic energy (kcal/mol), (e) van der Waal energy (kcal/mol), and (f) end-to-end distances (Å) for each chain; Figure S2: Mean square displacement (MSD) of PET/PEV blend at 2 ns, 5 ns, and 10 ns of last 10 ns production run. T represents the PET chain, and V represents the PEV chain; Figure S3: The number of contact atoms at 6 Å, which represents hydrophobic interaction; Figure S4: The number of hydrogen bonds formed between PET and PEV chains per time using a cut-off of 3.0 Å for CH…O and an angle of 180 ± 20 degrees for CH-H…O.
