# Supplementary material for: Prediction of the Glass Transition Temperature in Polyethylene Terephthalate/Polyethylene Vanillate (PET/PEV) Blends: A Molecular Dynamics Study
Source: Polymers (Basel). 2022 Jul 13;14(14):2858. doi: 10.3390/polym14142858 (PMC9317600; doi:10.3390/polym14142858)
Supplement: Supplementary file 1 [file polymers-14-02858-s001.zip › polymers-1774915-supplementary.pdf]

Supporting Material

Prediction of the Glass Transition Temperature in Polyethylene Terephthalate/Polyethylene Vanillate (PET/PEV) Blends: A Molecular Dynamics Study

Mattanun Sangkhawasi [1], Tawun Remsungnen [2,*], Alisa S. Vangnai [3], Phornphimon Maitarad [4] and Thanyada Rungrotmongkol [3,5,*]

[1] Program in Biotechnology, Faculty of Science, Chulalongkorn University, Bangkok 10330, Thailand; mattajung@gmail.com
[2] Faculty of Interdisciplinary Studies, Khon Kaen University, Nong Khai Campus, Nong Khai 43000, Thailand; rtawun@kku.ac.th
[3] Center of Excellence in Biocatalyst and Sustainable Biotechnology, Department of Biochemistry, Faculty of Science, Chulalongkorn University, Bangkok 10330, Thailand; alisa.v@chula.ac.th
[4] Research Center of Nano Science and Technology, Shanghai University, NO 99, Shangda Road, PO Box 111, Baoshan district, Shanghai 200444, China; pmaitarad@shu.edu.cn
[5] Program in Bioinformatics and Computational Biology, Graduate School, Chulalongkorn University, Bangkok 10330, Thailand; thanyada.r@chula.ac.th
* Correspondence: rtawun@kku.ac.th (T.R.); thanyada.r@chula.ac.th (T.R.); Tel.: +66-81499-2030 (T.R.); +66-2218-5426 (T.R.)

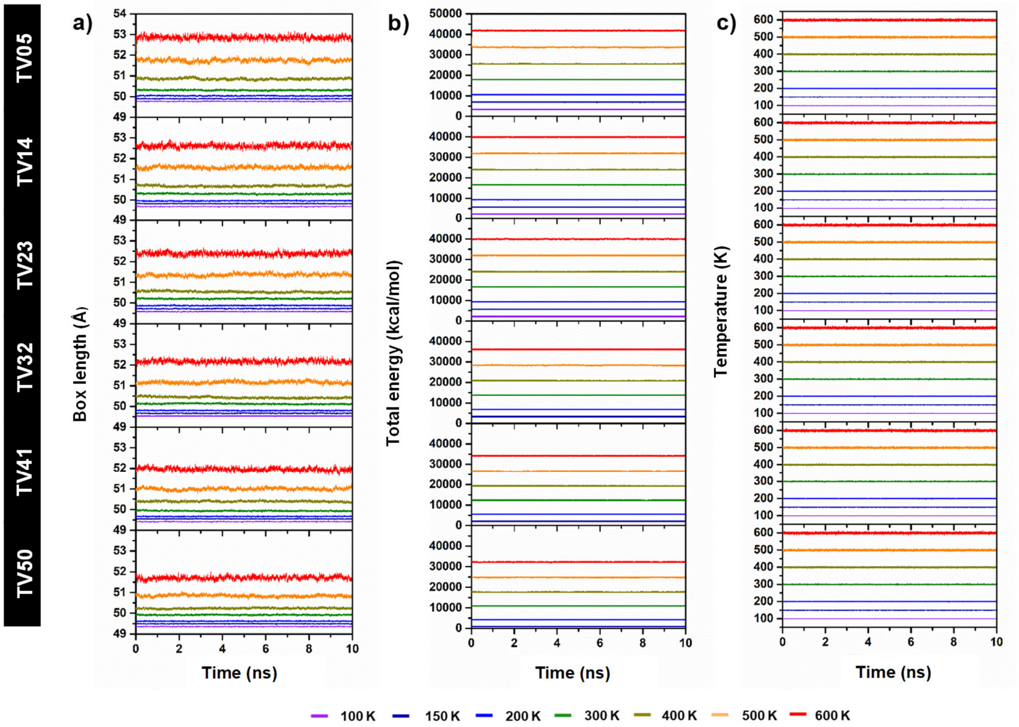

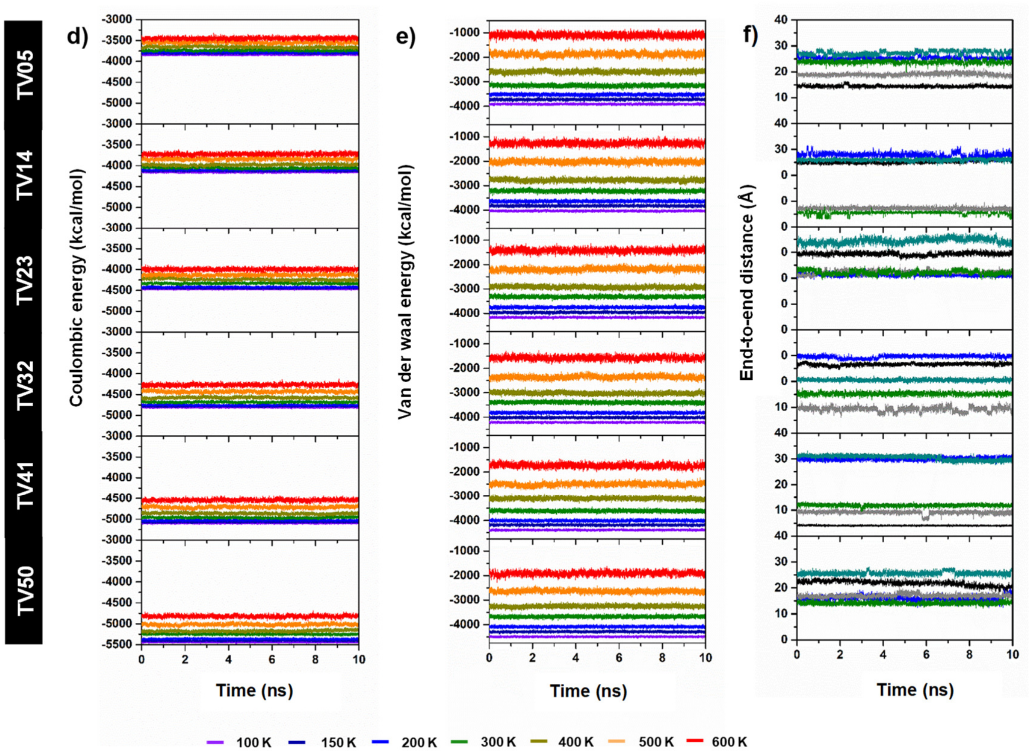

Figure S1. The investigation of system equilibrium (**a**) box length (Å) of production run from 1 to 10 ns, (**b**) temperature, (**c**) total energy profile (kcal/mol), (**d**) coulombic energy(kcal/mol), (**e**) van der Waal energy (kcal/mol) and (**f**) end-to-end distances (Å) for each chain.

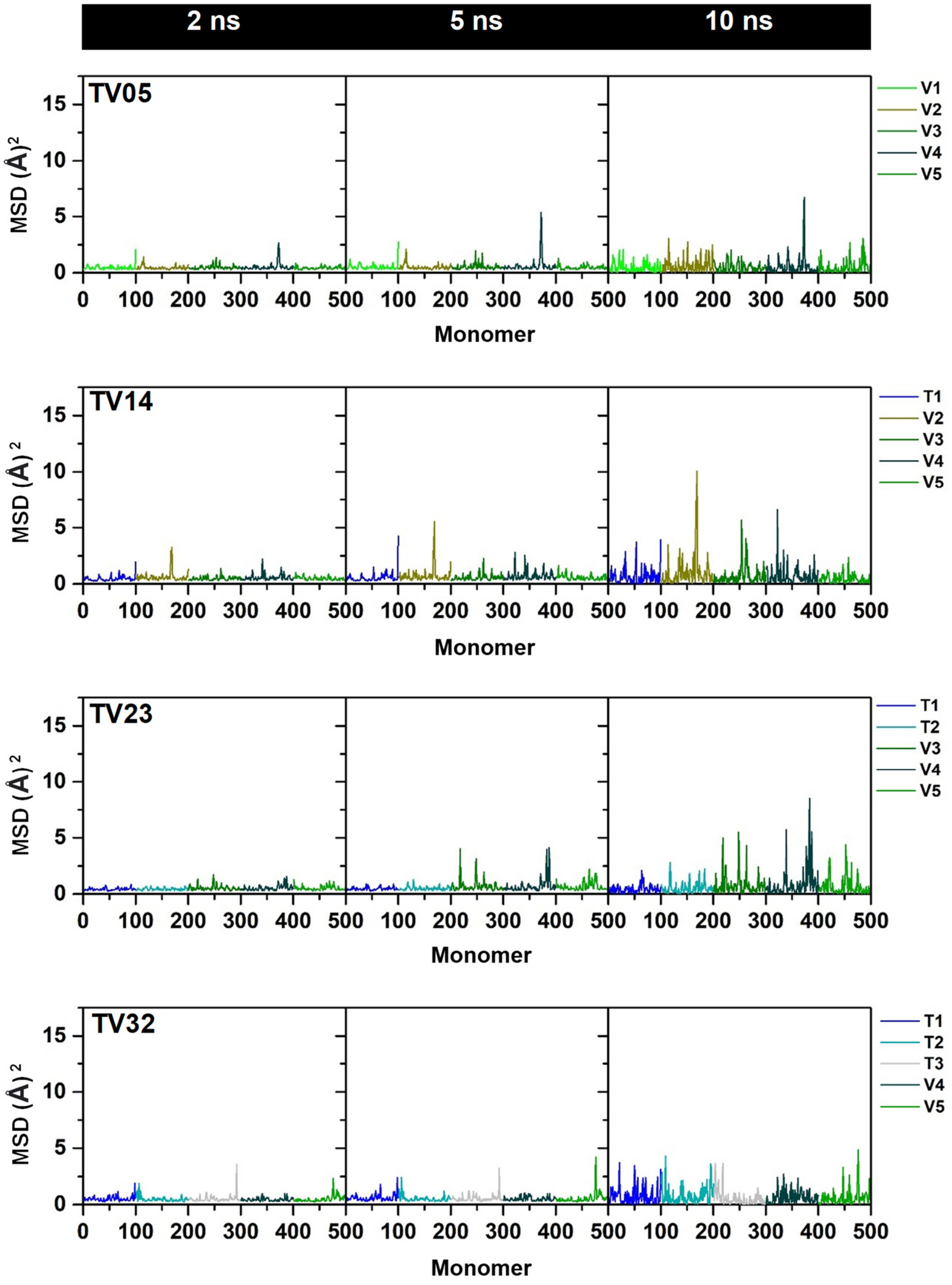

2 ns
5 ns
10 ns
TV05
V1
V2
V3
V4
V5
TV14
T1
V2
V3
V4
V5
TV23
T1
T2
V3
V4
V5
TV32
T1
T2
T3
V4
V5
MSD (Å)^2
Monomer
0
100
200
300
400
500
0
5
10
15

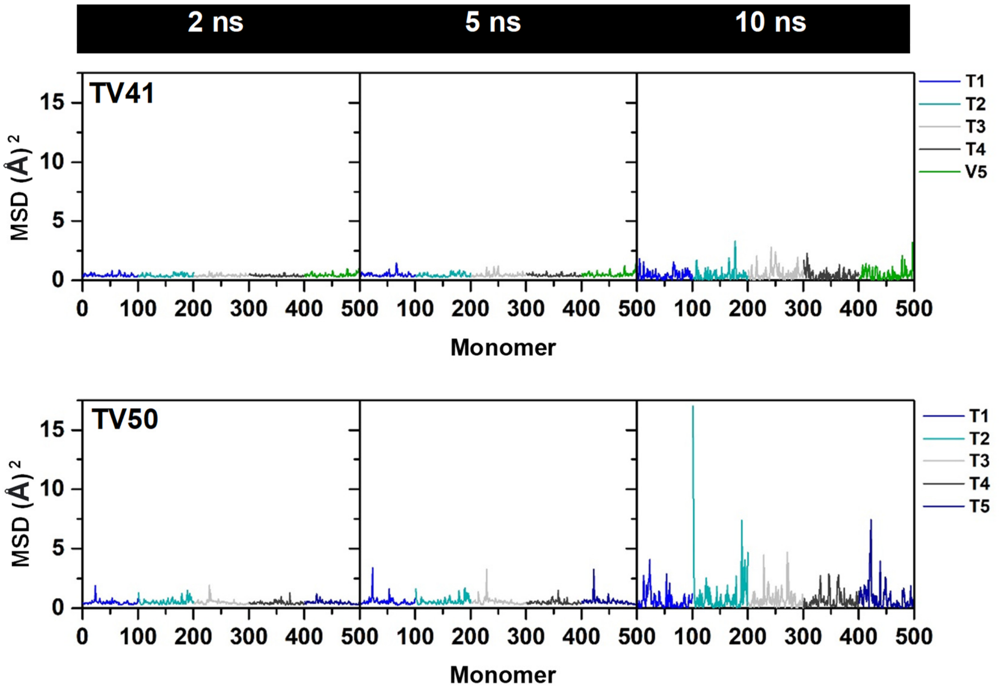

Figure S2. Mean square displacement (MSD) of PET/PEV blend at 2 ns, 5 ns, and 10 ns of last 10-ns production run. T represented PET chain and V represented PEV chain.

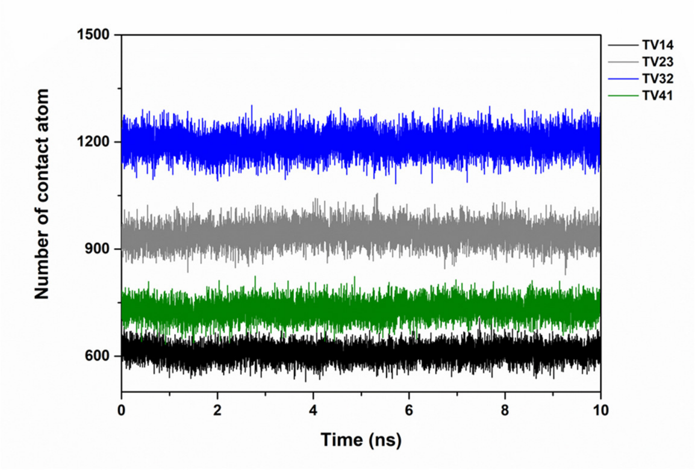

Figure S3. The number of contact atom at 6 Å, which represent hydrophobic interaction.

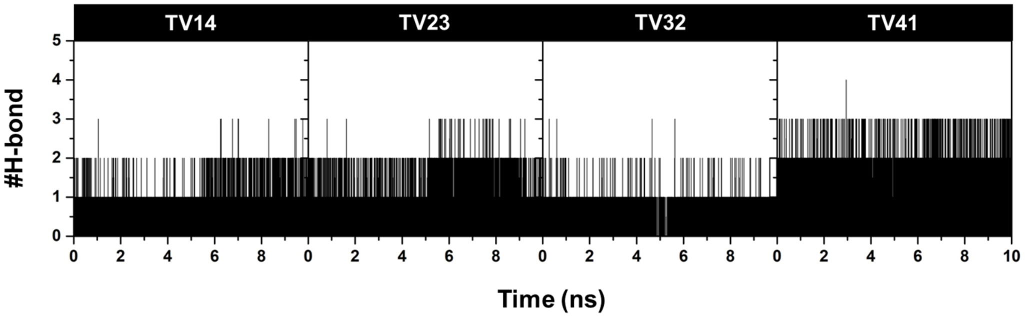

Figure S4. The number of hydrogen bonds formed between PET and PEV chains per time using a cut-off of 3.0 Å for CH…O and an angle of 180±20 degrees for CH-H…O.
